# Tuberculosis in Internationally Displaced Children Resettling in Harris County, Texas, USA, 2010–2015^1^

**DOI:** 10.3201/eid2608.190793

**Published:** 2020-08

**Authors:** Gabriella S. Lamb, Andrea T. Cruz, Elizabeth A. Camp, Michelle Javier, Jessica Montour, Tamisha Piper, Umair A. Shah, Jeffrey R. Starke

**Affiliations:** Baylor College of Medicine, Houston, Texas, USA (G.S. Lamb, A.T. Cruz, E.A. Camp, M. Javier, J.R. Starke);; US Committee for Refugees and Immigrants, Austin, Texas, USA (J. Montour);; Harris County Public Health Refugee Health Screening Program, Houston (T. Piper, U.A. Shah)

**Keywords:** tuberculosis and other mycobacteria, children, epidemiology, internationally displaced, refugee, asylee, tuberculin skin test, interferon-gamma release assay, Texas, United States, bacteria

## Abstract

US guidelines have recommended testing children emigrating from high tuberculosis-incidence countries with interferon-gamma release assays (IGRAs) or tuberculin skin tests (TSTs). We describe the Harris County (Texas) Public Health Refugee Health Screening Program’s testing results during 2010–2015 for children <18 years of age: 5,990 were evaluated, and 5,870 (98%) were tested. Overall, 364 (6.2%) children had ≥1 positive test: 143/1,842 (7.8%) were tested with TST alone, 129/3,730 (3.5%) with IGRA alone, and 92/298 (30.9%) with both TST and IGRA. Region of origin and younger age were associated with positive TST or IGRA results. All children were more likely to have positive results for TST than for IGRA (OR 2.92, 95% CI 2.37–3.59). Discordant test results were common (20%) and most often were TST+/IGRA– (95.0%), likely because of bacillus Calmette-Guérin vaccination. Finding fewer false positives supports the 2018 change in US immigration guidelines that recommends using IGRAs for recently immigrated children.

## Abstract 2

For testing immigrant children, research supports using interferon-gamma release assays rather than tuberculin skin tests.

The World Health Organization (WHO) has estimated that there were 1 million tuberculosis (TB) cases and 234,000 tuberculosis-related deaths among children in 2017 (*1*). An estimated additional 67 million children were infected with *Mycobacterium tuberculosis* (*2*). Testing of children emigrating from high- to low-incidence countries can provide benefit to individual patients and to the community by identifying patients for whom providing treatment would reduce the risk of progression to disease and thus decrease the reservoir for future contagious disease cases.

Not all recently arrived children are at equal risk for TB infection. Refugees, asylees, and victims of human trafficking (VHTs) may be at highest risk as the result of prolonged periods of displacement, undernutrition, poor sanitation, and poor access to medical care (*3*). In the United States, special immigrant visa (SIV) holders are predominantly children of persons from Afghanistan or Iraq who served as military translators and do not typically live in congregate settings (*4*,*5*), potentially placing them at lower risk for TB exposure. Those with parole classification most commonly emigrated from Cuba, a country with a low incidence of TB (*6**–**8*).

In 2018, the Centers for Disease Control and Prevention (CDC) updated the preimmigration guidelines for TB testing for immigrants to the United States, recommending that children 2–14 years of age who come from high TB-incidence countries (>20 cases/100,000 population) be tested with an interferon-gamma release assay (IGRA) rather than a tuberculin skin test (TST) (*9*). Previously, because of resource restrictions, TSTs were performed more commonly in many countries, and 9%–35% of refugee children tested positive (*10**–**15*). However, in a large study, almost two thirds of children with positive preimmigration TST results had negative IGRA results on postimmigration testing (*11*), indicating that many positive TST results likely were caused by prior vaccination with bacillus Calmette-Guérin (BCG).

Almost 10% of refugees, asylees, parolees, or SIV holders in the United States resettle in Texas, and almost 25% of those resettle in Harris County (which includes Houston) (*16*). Texas is also a human trafficking hub, with many internationally trafficked persons passing through the state (*17*). We describe the comparative epidemiology of positive TSTs and IGRAs in children of different immigration classifications cared for through the Harris County Public Health Refugee Health Screening Program.

## Methods

The Harris County Public Health Refugee Health Screening Program performs intake screening for TB, HIV, and pathogenic parasites and performs other routine laboratory screenings for all refugees, asylees, identified VHTs, parolees, and SIV holders resettling in the county. We performed a cross-sectional study of children 0–18 years of age who were evaluated by this program during January 1, 2010–December 31, 2015. We obtained demographic information, TB exposure history, symptom screening, and testing results from the Harris County Public Health Refugee Health Screening Program and the US Committee for Refugees and Immigrants (although the data originated from the Texas Department of State Health Services when the Texas Refugee Health Program resided there). All children were seen in a clinic run by the Harris County Public Health Refugee Health Screening Program. At this visit, demographic information and testing before immigration were obtained from the family and overseas records. Children not tested before immigration or who had no overseas records were tested during the clinic visit. Most of the children with a positive test for TB infection were evaluated at the Texas Children’s Hospital TB Clinic where additional testing and, if indicated, treatment were provided by 3 of the authors of this article (G.S.L., A.T.C., and J.R.S.).

Immigration classification was determined by the US Committee for Refugees and Immigrants. Immigration classifications are defined by US Citizenship and Immigration Services (Table 1) (*18**–**22*). We used WHO definitions for regions of origin (*23*). We predicted that refugees, asylees, and VHTs would be higher-risk groups for TB infection because they were more likely to have lived in congregate settings. We predicted the lower-risk groups to be parolees and SIVs because of their residence in lower TB-incidence nations and not living in congregate settings. Although overseas vaccination data were unavailable, we assumed that children were BCG immunized because most children emigrated from countries where universal BCG vaccination is practiced.

**Table 1:** Definitions of immigration classifications, United States

Immigration classification	Definition
Refugee	A person located outside the United States who demonstrates he or she was persecuted or has a fear of persecution because of race, religion, nationality, political opinion, or membership in a particular social group and is not firmly resettled in another country (*18*).
Asylee (asylum seeker)	Any person who meets the definition of a refugee and is already in the United States or seeking admission at a port of entry (*19*).
Parolee (Cuban and Haitian Family Reunification Parole Programs)	Persons from Cuba and Haiti who have family members who are US citizens or lawful permanent residents, who are able to come to the United States without waiting for immigrant visas to become available (*20*).
Special immigrant visa (holders	There are many categories; however, the children included in this study are children of Iraqi and Afghan translators who are interpreters who have worked with the US Armed Forces or under the chief of mission authority at the US embassy in Baghdad or Kabul (*21*).
Victim of human trafficking	A person who has been recruited, harbored, or transported for compelled labor or commercial sex acts through the use of force, fraud, or coercion (*22*).

Clinicians in the Harris County Public Health Refugee Health Screening Program performed initial TB testing. Providers were able to choose the type of TB testing used; they typically used the TST in children <5 years of age and an IGRA in children ≥5 years of age (*24*). TST results were considered positive if there was ≥10 mm of induration, unless the child was living with HIV or had contact with a person with pulmonary TB, in which case the threshold was ≥5 mm of induration (*25*). The main IGRA used was the T-SPOT.*TB* (Oxford Immunotec, https://www.tspot.com), for which a result was defined as positive if ≥8 spots were noted in either well (*24*). A positive QuantiFERON Gold-In Tube (QIAGEN, https://www.qiagen.com) result was defined as an antigen-nil value of ≥0.35 IU/mL (*24*).

We classified children with positive test(s) for infection as TB infected, likely TB uninfected, or having TB disease. These classifications were determined by 2 authors (ATS and JRS) at the time each child was seen. In the first 2 categories, children had normal physical examinations and 2-view chest radiographs. We classified children as having TB infection if they had a positive IGRA result (IGRA+), if they had a positive TST result (TST+) and no IGRA was done (not all TST+ children had IGRAs performed), or if the IGRA result was indeterminate/invalid. We typically defined children as being likely uninfected if they were TST+/IGRA–, had normal physical examination findings and normal chest radiographs, and had no known contacts with TB disease or if they had negative tests with normal physical examination findings and chest radiographs. However, we did not classify all children who were TST+/IGRA– as being uninfected; some of these children were classified as having TB infection, most commonly because of young age (<2 years). TB disease was diagnosed in children who had clinical, physical examination, or radiographic findings consistent with TB disease (*26*).

We created 3 models to determine which factors were independently associated with positive tests of infection: a positive TST result, a positive IGRA result, and any positive TB test result (TST or IGRA). We compared demographic characteristics and other categorical variables among the higher-risk and lower-risk groups for statistically significant differences by the χ^2^ test or Fisher exact test for dichotomous variables and Wilcoxon rank-sum or Kruskal-Wallis test for continuous variables. We included any factor with a p value ≤0.25 in the binary regression model. We created the final model using a backward-step approach. To assess secular trends in usage and positivity, we analyzed monthly totals using linear regression and the Wilcoxon signed-rank test. A p value <0.05 was considered significant.

We conducted all analyses using the SPSS Statistics 25 (IBM, https://www.ibm.com). We obtained institutional review board approval from the Harris County Public Health Department and Baylor College of Medicine (Houston, TX, USA).

## Results

**Figure 1:**
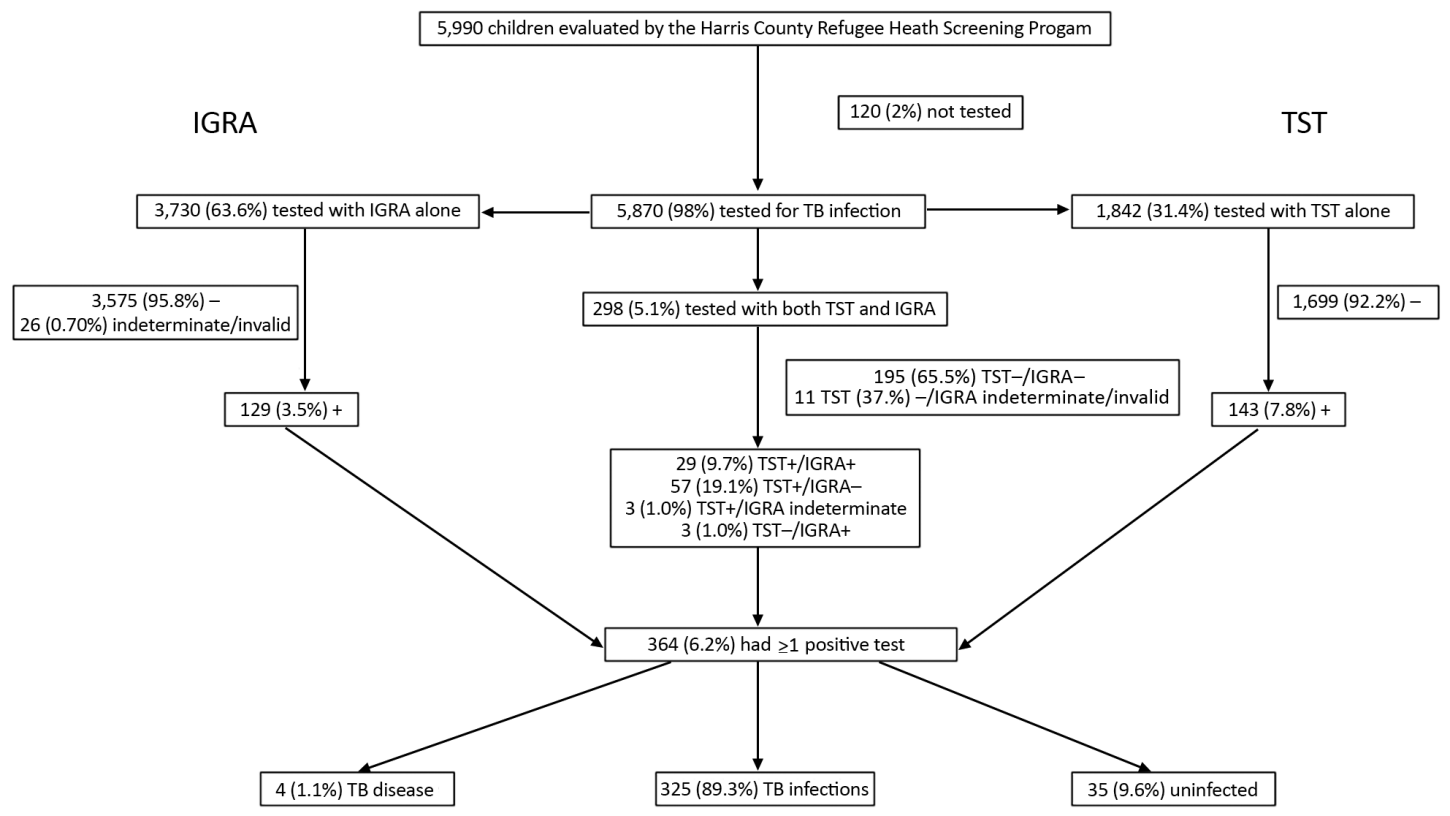
Consort diagram of TST and IGRA results in internationally displaced children over a 6-year period, Harris County, Texas, USA, 2010–2015. The percentage reported for TB disease, infection, and uninfected are the percentage of persons who had ≥1 positive test. IGRA, interferon gamma release assay; TST, tuberculin skin test; TB, tuberculosis; +, positive; –, negative.

During the study period, the Harris County Public Health Refugee Health Screening Program evaluated 5,990 children (Table 2), 98% (5,870) of whom received ≥1 test of TB infection (Table 3): IGRA, 3,730 (63.5%); TST, 1,842 (31.4%); both TST and IGRA, 298 (5.1%). In the TST and IGRA group, 206 (69.1%) were TST–/IGRA–, 29 (9.7%) TST+/IGRA+, 57 (19.1%) TST +/IGRA–, 3 (1.0%) TST+/IGRA indeterminate/invalid, and 3 (1.0%) TST–/IGRA+ (Figure 1). Discrepant test results occurred in 60 (20.1%) children tested with both TST and IGRA.

**Table 2:** Demographic variables of internationally displaced children, Harris County, Texas, USA, 2010–2015*

Category	Variable	Immigration classification	p value

Higher-risk groups				Lower-risk groups	
Refugee, n = 4,246	Asylee, n = 173	VHT, n = 16	Parolee, n = 851	SIV holder, n = 704
Demographics	Age, y, median (IQR)	8 (4–12)	9 (5–12)	11.5 (7–15)		9 (5–13)	4 (2–8)	<0.001†
	Female sex	2,025 (47.7)	81 (46.8)	7 (43.8)		404 (47.5)	304 (43.2)	0.281‡
WHO region of origin	Eastern Mediterranean	1,414 (33.3)	89 (51.4)	0		0	704 (100.0)	<0.001‡
	Southeast Asia	1,416 (33.3)	7 (4.0)	1 (6.3)		0	0	
	Africa	1,202 (28.3)	40 (23.1)	0		0	0	
	America	183 (4.3)	31 (17.9)	12 (75.0)		851 (100.0)	0	
	Western Pacific	18 (0.4)	2 (1.2)	1 (6.3)		0	0	
	European	13 (0.3)	4 (2.3)	2 (12.5)		0	0	
Underlying conditions	HIV infected	22 (0.5)	0	0		1 (0.1)	2 (0.3)	<0.001‡
	Pathogenic parasites detected in feces	328 (17.1)	18 (11.3)	2 (12.5)		98 (12.5)	115 (17.9)	<0.001‡

*Values are no. (%) except as indicated. Percentages reflect those for whom the information was available. IQR, interquartile range; SIV, special immigrant visa; VHT, victim of human trafficking; WHO, World Health Organization. †p value using Kruskal-Wallis test. ‡p value using χ^2^ test.

**Table 3:** Tests for TB infection performed and test results by immigration classification in migrant children, Harris County, Texas, USA, 2010–2015*

Category	Immigration classification	OR (95% CI)	p value†

Higher-risk groups				Lower-risk groups	
Refugee, n = 4,246	Asylee, n = 173	VHT, n = 16	Parolee, n = 851	SIV holder, n = 704
Test(s) performed								
TST alone	1,381 (32.5)	30 (17.3)	1 (6.3)		168 (19.7)	300 (42.6)	1.08 (0.96–1.23)	<0.001
IGRA alone	2,558 (60.2)	137 (79.2)	15 (93.8)		642 (75.4)	349 (49.6)	1.12 (0.99–1.26)	
TST and IGRA	204 (4.8)	4 (2.3)	0		22 (2.6)	29 (4.1)	1.45 (1.06–1.98)	
**Result**								
TST positive	196/1,589 (12.3)	4/35 (11.4)	0		7/190 (3.7)	25/329 (7.6)	2.14 (1.45–3.15)	<0.001
IGRA positive	142/2,819 (5.0)	5/141 (3.5)	0		5/664 (0.8)	9/389 (2.3)	3.84 (2.21–6.68)	<0.001
Total positive tests	311 (7.3)	9 (5.2)	0		10 (1.2)	34 (4.8)	2.67 (1.94–3.68)	<0.001

*Values are no. (%) or no. positive/no. tested (%)** except as indicated. **IGRA, interferon gamma release assay; OR, odds ratio; SIV, special immigrant visa; TST, tuberculin skin test; VHT, victim of human trafficking. †p value using χ^2^ test.

Overall, 364 children (6.2%) had ≥1 positive TB test. Among these children, 325 (89.3%) received diagnoses of TB infection, 35 (9.6%) were considered likely uninfected, and 4 (1.1%) received diagnoses of TB disease (Figure 1). The 35 children with a positive test who were considered likely uninfected were all TST+/IGRA–. In addition, 22 (38.6%) children who were TST+/IGRA– were classified as having TB infection, typically earlier in the study period because of young age, variability in provider practice, or both (Figure 1). 

The Texas Children’s Hospital Tuberculosis Clinic in Houston cares for most of children in Harris County with TB disease. According to a chart review, none of the children who had TB infection or who were considered likely uninfected had TB disease developed during the following 4–9 years (2,427 person-years of follow-up). Furthermore, we cross-referenced the public health records for Harris County and found that none of these children had been reported to have TB disease develop.

We found 3 factors to be associated with a either a positive TST or IGRA: region of origin, age group, and HIV status. Immigration classification was associated with a positive TB test result on univariate analysis, but this association did not hold true on multivariate analysis. Children, irrespective of immigration classification and from all regions and age groups, had greater odds of having a positive TST result than a positive IGRA result (OR 2.92, 95% CI 2.79–3.59).

### Immigration Classification

On univariate analysis, irrespective of test performed, children determined to have a higher-risk immigration classification had nearly 3 times the odds of having a positive TST or IGRA compared with those with lower-risk immigration classifications (OR 2.68, 95% CI 1.94–3.68). Specifically, children with higher-risk immigration classifications had twice the odds of having a positive TST (OR 2.14, 95% CI 1.45–3.15) and nearly 4 times the odds of having a positive IGRA (OR 3.84, 95% CI 2.21–6.68) compared with children with lower-risk immigration classifications (Table 3). These differences were not seen on multivariate analysis.

All children, regardless of immigration classification, had greater odds of a positive result for TST compared with IGRA (OR 2.92, 95% CI 2.79–3.59). This difference was more pronounced among those with lower-risk classification (OR 4.81, 95% CI 2.54–9.10) than those with higher-risk classification (OR 2.68, 95% 2.15–3.35).

### Region of Origin

**Figure 2:**
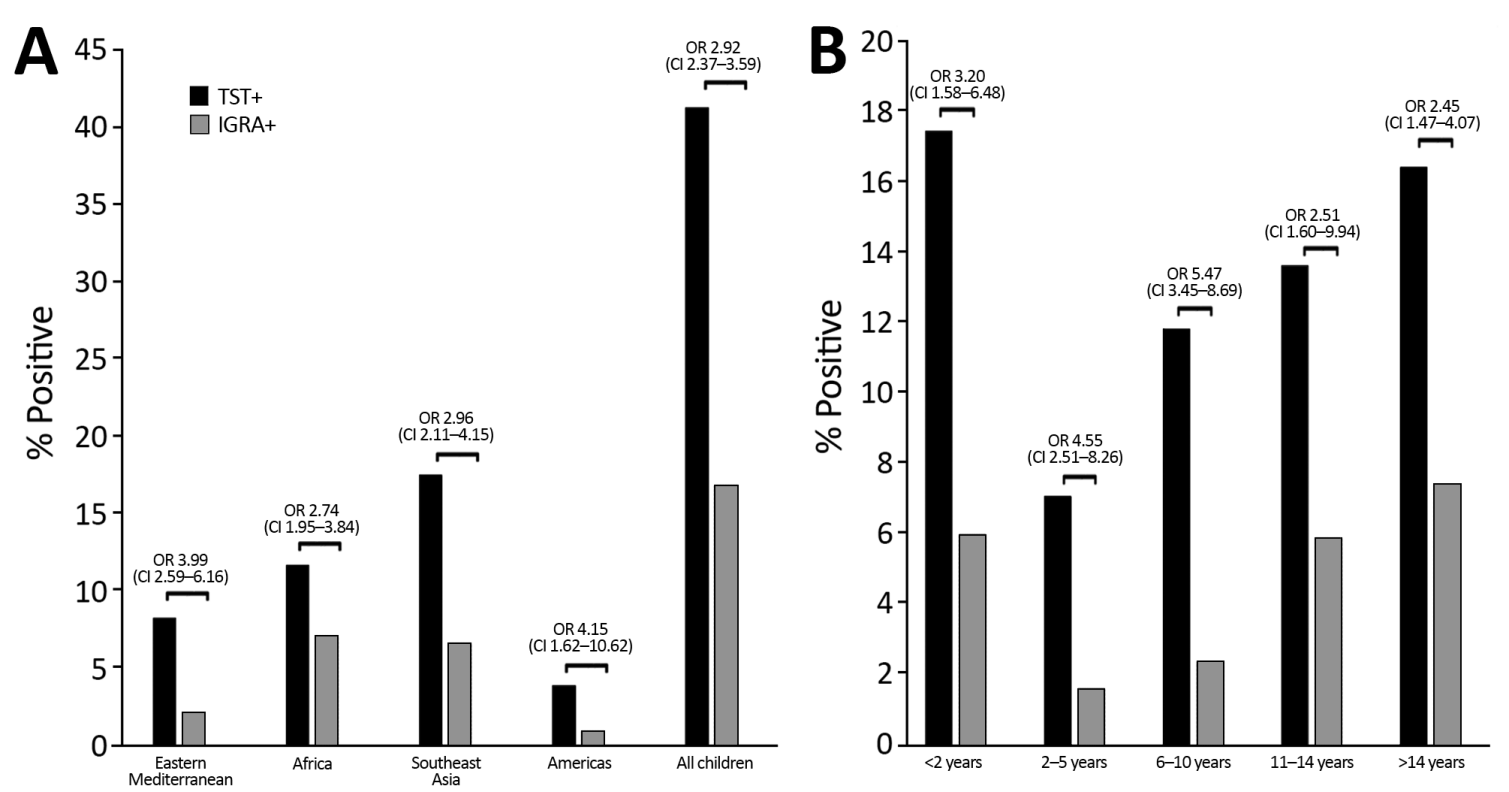
Comparison of TST and IGRA results (using IGRAs as reference) in internationally displaced children over a 6-year period, Harris County, Texas, USA, 2010–2015. A) By location; B) by age. Brackets indicate ORs and 95% CIs between categories. IGRA, interferon gamma release assay; OR, odds ratio; TST, tuberculin skin test.

On multivariate analysis, region of origin was a notable correlate for a positive test of TB infection. TST and IGRA positivity varied by region of origin. Using children from Southeast Asia as a reference group, we found that children from eastern Mediterranean countries (adjusted odds ratio [aOR] 0.48, 95% CI 0.33−0.70) and the Americas (aOR 0.19, 95% CI 0.09–0.39) had reduced odds for a positive TST result compared with children from Southeast Asia. Similarly, children from eastern Mediterranean countries (aOR 0.34, 95% CI 0.21−0.53) and the Americas (aOR 0.12, 95% CI 0.06–0.25) had reduced odds for a positive IGRA result compared with children from Southeast Asia (Table 4). Using the IGRA result as the reference, we found the odds of having a positive TST result to be greater in children from eastern Mediterranean countries (OR 3.99, 95% CI 2.59–6.16) and the Americas (OR 4.15, 95% CI 1.62–10.62) (Figure 2, panel A).

**Table 4:** Factors associated with a positive result for TST, IGRA, or both in 5,870 migrant children, Harris County, Texas, USA, 2010–2015*

Characteristic	TST model				IGRA model				TST and IGRA combined model		
	No.†	aOR (95% CI)	p value		No.†	aOR (95% CI)	p value		No.†	aOR (95% CI)	p value
Age, y											
<2	53	Referent			10	Referent			55	Referent	
2–5	75	0.40 (0.26–0.61)	<0.001		13	0.26 (0.11–0.60)	0.002		81	0.36 (0.24–0.54)	<0.001
6–10	43	0.72 (0.45–1.07)	0.25		35	0.41 (0.20–0.85)	0.02		74	0.28 (0.18–0.42)	<0.001
11–14	34	0.68 (0.39–1.21)	0.19		56	1.05 (0.52–2.12)	0.90		85	0.55 (0.37–0.83)	0.004
>14	27	0.77 (0.44–1.35)	0.36		47	1.35 (0.66–2.77)	0.41		69	0.72 (0.48–1.10)	0.13
Region of origin											
Southeast Asia	99	Referent			62	Referent			149	Referent	
E. Mediterranean	74	0.48 (0.33–0.70)	<0.001		30	0.34 (0.21–0.53)	<0.001		98	0.44 (0.33–0.59)	<0.001
Africa	49	0.69 (0.45–1.07)	0.10		61	1.02 (0.70–1.49)	0.91		100	0.81 (0.61–1.09)	0.17
Americas	10	0.19 (0.09–0.39)	<0.001		8	0.12 (0.06–0.25)	<0.001		17	0.14 (0.08–0.23)	<0.001
HIV positive	5	2.99 (1.01–8.87)	0.049		2	–	–		7	5.57 (2.23–13.90)	<0.001

*aOR, adjusted odds ratio; E., Eastern; IGRA, interferon gamma release assay; TST, tuberculin skin test; –, numbers too small to enable performance of these tests. †Number in group with a positive TB test result.

### Age Group

TST and IGRA positivity varied by age group. Using children <2 years of age as the reference group, on multivariate analysis, we found that children 2–5 years of age had reduced odds for a positive TST result (aOR 0.40, 95% CI 0.26–0.61) and children 2–10 years of age had reduced odds for a positive IGRA result (2–5 years, aOR 0.26, 95% CI 0.11–0.60; 6–10 years, aOR 0.41, 95% CI 0.20–0.85) (Table 4).

Using the IGRA result as the reference, we found that all children, regardless of age, had greater odds of having a positive TST than a positive IGRA (OR 2.92, 95% CI 2.79–3.59). Children 6–10 years of age had the greatest odds of having a positive TST result compared with a positive IGRA result (OR 5.47, 95% CI 3.45–8.69) (Figure 2, panel B).

### HIV Infection

On multivariate analysis, we found that children living with HIV had 3 times the odds for a positive TST result compared with children who were HIV uninfected (aOR 2.99, 95% CI 1.01–8.87). HIV infection was not associated with a positive IGRA result (Table 4).

## Discussion

In the United States, 66% of reported TB cases occur among foreign-born persons, a rate 13 times higher than for persons born in the United States (*27*). Previous studies, mostly using the TST, found a prevalence of TB infection of 9%–35% among refugee children (*10**–**15*). However, positive results were less common in our cohort, likely because of the variety of immigration classifications included and expanded IGRA use.

We found that the prevalence of positive results for tests of TB infection varied by region of origin and age and that all children in the study had greater odds of a positive result from TST than from IGRA. Immigration classification was not associated with positive results for TB infection. We also found discordance between TST and IGRA results across the pediatric age spectrum, suggesting that the effect of BCG vaccination on TST positivity may be more prolonged than typically expected (*28*). In addition, potentially confounding our results, children who received TSTs before and after immigration may have had immunologic boosting, resulting in falsely positive TSTs and IGRAs (*29*).

Region of origin was a notable risk factor for positive TB test results. Children from Southeast Asia had greater odds of having a positive result for TST, IGRA, or both than did children from eastern Mediterranean countries and the Americas, but we found no difference for these children compared with those from Africa. This finding is consistent with known epidemiologic risk factors: the prevalence of TB disease is high in countries in Africa and Southeast Asia, and Southeast Asia has a higher prevalence of TB disease than eastern Mediterranean countries and Cuba (where most children from the Americas region originated) (*30*).

Age was another noteworthy risk factor for positive TB test results. Children <2 years and >14 years of age had a higher prevalence of positive results for TST, IGRA, or both compared with children 2–14 years of age. Older children (>14 years of age) in our cohort had increased prevalence of positive TB test results by both the TST and IGRA, which more likely represented true TB infection because of the children’s greater time outside the home and cumulative exposure to adults with infectious pulmonary TB (*30**–**34*). The higher frequency of positive tests in children <2 years of age, on the other hand, is more difficult to explain. Higher TST positivity in children <2 years of age likely represented greater cross-reactivity with BCG, given the temporal proximity to vaccination or potential boosting if children had serial TSTs performed (before and after immigration). However, BCG immunization cannot explain the increased prevalence of positive IGRA results in these young children. Furthermore, these data contradict previous studies that demonstrate that older children are more likely to have TB infection (*30**–**34*). One possible explanation is that IGRA-positive children <2 years of age in our cohort had more prolonged exposure to an adult family member with infectious pulmonary TB in the home, because very young children spend more time in the home than their school-aged counterparts. To date, IGRAs have not been used routinely for the diagnosis of TB infection in children <2 years of age because of a paucity of data on test performance (*35*).

Most previous estimates of the prevalence of TB infection in immigrant children used the TST as the test of choice, given an initial paucity of pediatric data and the scarcity and cost of IGRAs. Our findings paralleled the results of a recent study using IGRA testing, which estimated that 5.6% of immigrant children had TB infection, compared with previous estimates of 22% based on TST testing (*36*). American Academy of Pediatrics guidelines currently recommend IGRA use down to 2 years of age (*35*); some experts recommend using IGRAs in children as young as 1 year of age. Use of IGRAs rather than the TST would likely reduce false positive tests and allow for TB infection therapy to be targeted to those who would most benefit.

The CDC does not recommend tiered testing (that is, obtaining an IGRA if a TST result is positive) for TB infection. However, at times, the initial test of infection, selected either by choice or by necessity, is not the optimal test, particularly for a BCG-immunized child. In our study, all children had greater odds of having a positive TST result compared with a positive IGRA test regardless of immigration classification, region of origin, or age. In addition, we had almost 300 children in whom both TSTs and IGRAs were obtained, of whom 20% had discordant results, mostly TST+/IGRA–. Our findings are consistent with a prior study that demonstrated that for BCG-immunized children who have immigration-related testing, false positive TST results are common, and IGRAs should be the tests of choice for this patient population (*10*).

Discordance between TST and IGRA results was seen in all age groups. Although false-positive results are an expected limitation of TST use among BCG-vaccinated children, the impact of BCG vaccine in causing falsely positive TST results has typically been thought to be temporally related to BCG vaccine receipt, which in most countries is a single dose immediately after birth. However, discordant TST and IGRA results in older children suggest either that the effect of some strains of BCG on the TST result lasts longer than previously recognized (*28*) or that the effect of nontuberculous mycobacteria infections that also can cause a falsely positive TST result may be underestimated in children from developing nations. A third possibility is that immunologic boosting as a result of repeat skin testing before and after immigration may have resulted in false positive TSTs (*29*). Concerns about false positive TST results are considered in updated 2018 guidelines (*9*) for TB testing before immigration, thereby suggesting use of IGRAs for all persons ≥2 years of age.

Our study has limitations. Although we have follow-up data from the Texas Children’s Hospital TB clinic for most children evaluated for a positive test of TB infection, we do not have data for some children evaluated at other clinics or for children whose test for TB infection was negative. BCG vaccination status was not routinely documented; thus, the presumption of false-positive TSTs secondary to cross-reactivity with BCG assumes that most children were BCG-immunized. We assumed BCG vaccination because this vaccination is recommended in the national immunization programs of 95% of countries from which these children emigrated (*37*). HIV infection was rare in our cohort, precluding drawing meaningful conclusions regarding positive results for TST, IGRA, or both in HIV-infected children from our data. Finally, these data may not be generalizable to all immigrant children relocating to the United States because this study included predominantly children from TB high-burden countries (*9*).

In summary, the prevalence of positive TB test results in this cohort of children was lower than previously reported, and TB disease was rare. The lower prevalence of positive tests of TB infection in this childhood population likely stems from the predominant use of IGRA testing. The TST and IGRA results are frequently discrepant, particularly among those with lower-risk immigration classification, younger children who have received a BCG vaccine, and those from lower-burden countries.

As a result of these data and our experience, we advocate for other health jurisdictions to implement the routine use of IGRA testing for all children, regardless of immigration classification, region of origin, or age, who are evaluated as part of the immigration process to the United States. We also advocate for use of confirmatory IGRA testing in BCG-immunized children with no known TB contacts who have positive TST results. Use of IGRA as opposed to TST in BCG-immunized children would reduce false-positive test results and enable TB infection therapy to be targeted to those who would most benefit.
